# A Virtual Agent to Support Individuals Living With Physical and Mental Comorbidities: Co-Design and Acceptability Testing

**DOI:** 10.2196/12996

**Published:** 2019-05-30

**Authors:** Katherine Easton, Stephen Potter, Remi Bec, Matthew Bennion, Heidi Christensen, Cheryl Grindell, Bahman Mirheidari, Scott Weich, Luc de Witte, Daniel Wolstenholme, Mark S Hawley

**Affiliations:** 1 School of Health and Related Research The University of Sheffield Sheffield United Kingdom; 2 Centre for Assistive Technology and Connected Healthcare The University of Sheffield Sheffield United Kingdom; 3 Collaboration for Leadership in Applied Health Research and Care Yorkshire and Humber Royal Hallamshire Hospital National Institute of Health Sheffield United Kingdom; 4 Lab4Living Art and Design Research Centre Sheffield Hallam University Sheffield United Kingdom; 5 Department of Computer Science The University of Sheffield Sheffield United Kingdom; 6 Department of Psychology The University of Sheffield Sheffield United Kingdom

**Keywords:** COPD, chronic obstructive pulmonary disease, mental health, comorbidity, chronic illness, self-management, artificial intelligence, virtual systems, computer-assisted therapy, chatbot, conversational agent

## Abstract

**Background:**

Individuals living with long-term physical health conditions frequently experience co-occurring mental health problems. This comorbidity has a significant impact on an individual’s levels of emotional distress, health outcomes, and associated health care utilization. As health care services struggle to meet demand and care increasingly moves to the community, digital tools are being promoted to support patients to self-manage their health. One such technology is the autonomous virtual agent (chatbot, conversational agent), which uses artificial intelligence (AI) to process the user’s written or spoken natural language and then to select or construct the corresponding appropriate responses.

**Objective:**

This study aimed to co-design the content, functionality, and interface modalities of an autonomous virtual agent to support self-management for patients with an exemplar long-term condition (LTC; chronic pulmonary obstructive disease [COPD]) and then to assess the acceptability and system content.

**Methods:**

We conducted 2 co-design workshops and a proof-of-concept implementation of an autonomous virtual agent with natural language processing capabilities. This implementation formed the basis for video-based scenario testing of acceptability with adults with a diagnosis of COPD and health professionals involved in their care.

**Results:**

Adults (n=6) with a diagnosis of COPD and health professionals (n=5) specified 4 priority self-management scenarios for which they would like to receive support: at the time of diagnosis (*information provision*), during acute exacerbations (*crisis support*), during periods of low mood (*emotional support*), and for general self-management (*motivation*). From the scenario testing, 12 additional adults with COPD felt the system to be both acceptable and engaging, particularly with regard to internet-of-things capabilities. They felt the system would be particularly useful for individuals living alone.

**Conclusions:**

Patients did not explicitly separate mental and physical health needs, although the content they developed for the virtual agent had a clear psychological approach. Supported self-management delivered via an autonomous virtual agent was acceptable to the participants. A co-design process has allowed the research team to identify key design principles, content, and functionality to underpin an autonomous agent for delivering self-management support to older adults living with COPD and potentially other LTCs.

## Introduction

### Comorbid Mental Health and Physical Long-Term Conditions

In the past decade, it has been acknowledged in research [1], government policy [2] and clinical practice [3] that physical conditions, such as respiratory and cardiovascular disease, and mental health conditions, including anxiety and depression, frequently coexist. It is estimated that 30% of those with 1 or more physical long-term conditions (LTCs) in England will have a comorbid mental health condition [3,4]. This is particularly the case for patients with a diagnosis of chronic obstructive pulmonary disease (COPD), a patient population that has a higher prevalence of anxiety and depression [5] and which is up to 10 times more likely to experience panic attacks than the general population [6]. These mental health conditions are distressing, have a clinically significant impact on health-related quality of life, and can lead to further health complications and greater consumption of costly health care [1,3,7]. For those living with COPD, management of their condition means maintenance of mental health as much as of physical health.

Unfortunately, the presence of mental health conditions in individuals with a physical LTC can go undetected and untreated. The addition of a physical disease process can compound mental health problems [8-10], and many individuals with LTCs do not have access to mental health care once clinically identified. Despite mental health problems accounting for 23% of the disease burden in the United Kingdom, only 11% of the UK National Health Service (NHS) budget is allocated to mental health services [11]. The high demand for services and a lack of trained health professionals able to offer quality care mean waiting times can be long [12,13]. Accordingly, the issues of management of both physical and mental health for patients with comorbidities is an imperative for research, policy, and clinical practice.

### Self-Management

Patient health care is increasingly moving into the community, with greater emphasis placed on supporting patients to *take control* of their own health (patient activation [14,15]). Self-management of health includes maintaining positive emotional well-being, eating a healthy diet, exercising, optimizing medication, monitoring symptoms, coping with changes in symptoms, and knowing when to seek help [15]. To do this, patients require techniques to draw upon and support to develop the self-confidence and skills required. A review of 228 systematic reviews of self-management interventions indicates that there is little consistent evidence for the appropriate duration, mode of delivery, or specific components of such interventions [16]. Psychological approaches that include techniques such as relaxation training, mindfulness, motivational interviewing, and cognitive restructuring appear to be of benefit in helping patients improve their self-management skills [16]. In addition, health education provision, biofeedback techniques, and physical activity have been found to improve emotional well-being for patients with a range of LTCs [15,16]. Interactive Web-based self-management programs and telehealth initiatives could be particularly useful elements of self-management support for patients, irrespective of the diagnosis [15]. Regardless of the actual techniques and tools used, it is clear that any truly comprehensive and successful self-management intervention for LTCs must address mental health as well as physical health and should see the care of the 2 as forming a holistic *care package* for the patient [1].

### Technology and Health Care

Commonplace digital technology consumer products such as mobile phones, tablets, laptops, and wearable devices have the potential to support and expand the delivery of health services in a more accessible and efficient manner than traditional face-to-face, 9 am to 5 pm, weekday delivery of care. Patients can use these products to access, for example, Web-based health information, self-help materials, electronic therapies, blended care, peer support, and one-to-one consultations [17]. For some patients, the greater convenience, accessibility, and availability of digital services outweigh the advantages of face-to-face contact with health professionals [18]. However, for current digital health technology to be evaluated as engaging, as acceptable by patients, and as clinically effective as traditional face-to-face delivery of care, a blended or guided approach, which includes aspects of face-to-face or telephone support, is recommended rather than the use of digital tools in isolation from human contact [19-21]. It is possible that more sophisticated digital health interventions may address these shortcomings if they can replicate key elements of person-to-person interaction.

### Artificial Intelligence and the Provision of Therapy

A promising approach to the development of more sophisticated digital health services lies in the use of artificial intelligence (AI) to build computer systems that are able to deliver therapeutic services with a certain degree of autonomy [22]. In such systems, the role of AI is to process the user’s input data (usually in the form of written or spoken natural language utterances) and then to select or construct the corresponding appropriate responses.

In some respects, the use of AI-based systems in this way represents a natural development: as conventional psychotherapy typically relies on a series of language-based interactions with a therapist, any system that can input and output the symbols that constitute that language—in other words, that has the potential to communicate with the patient—represents, in theory at least, a platform for the delivery of that therapy. Digital computers (and networks of digital computers, such as the Web) constitute one such platform, and as researchers in AI have devoted much effort over the years to emulating human natural language processing on digital computers, the use of AI to deliver therapy readily suggests itself. (Indeed, one of the earliest and most famous AI systems, ELIZA, dating from the 1960s, emulated, albeit in a somewhat tongue-in-cheek fashion, a *talking cure* therapist.) Moreover, computer programs and services promise to overcome some of the physical limitations of a human therapist: they can be duplicated rapidly and almost without limit, and they are not fixed to a physical location and never tire of operation, being available (almost) wherever and whenever they are needed.

In recent years, several such *autonomous virtual agents* have been developed to support the delivery of psychological therapies, incorporating automated responses to text-based chat [23,24]. However, being text based, these therapies lack the naturalness and immediacy of spoken language and can exclude those not skilled in typing, and it can be difficult to convey the subtleties of emotion or attitude. It seems likely that more inclusive and sophisticated therapeutic agents will have to incorporate spoken language interfaces.

### Avachat: A Virtual Agent for Long-Term Condition Self-Management

The aim of the work presented here was to explore the acceptability of an autonomous agent for supporting people with comorbid physical LTCs and mental health problems. The exemplar LTC chosen in this instance was COPD because of the high reported co-occurrence of common mental health conditions. We hypothesized a virtual agent system, called *Avachat* (a portmanteau of *avatar* and *chat*, but also a serendipitous near-homophone of *have a chat*), that would offer users acceptable support and guidance based on self-management principles [15]. This system was to be structured around a persona or character, christened *Ava*, which would act as a focus for the user’s interactions with the system and with which the user would—perhaps—form something akin to a therapeutic relationship. We imagined that Ava would come to personify the support mechanisms, with a visible (onscreen) and audible presence, and that its users would interact with it through natural language, but beyond this, we made few assumptions as to its nature, intending that this should emerge during a co-design process.

## Methods

### Design

Working with people with lived experience of COPD and health care professionals and using existing paper-based self-management materials as a starting point, we had the following objectives:

1. To co-design the *core functionality* of the system by identifying common problems faced when (self-)managing COPD, along with the solutions that were thought most appropriate or useful. These problems were then prioritized according to their impact on everyday life.
2. To elicit prospective users’ *operational requirements* for the system, including interface features and visual appearance and delivery modalities, and, based on these, the co-design as far as possible of the corresponding features of the system.
3. To develop a prototype implementation that would meet the core functionality and operational requirements as comprehensively as possible given the constraints on resources (time, personnel, and available technology). The prototype was to be developed rapidly to allow its use in a subsequent iteration of the co-design process.
4. To test the *acceptability* of the design concepts with a wider group of participants.

These methods were adapted from the work of the UK National Institute for Health Research (NIHR) Collaboration for Leadership in Applied Health Research and Care Yorkshire and Humber [25] *Translating Knowledge into Action* theme. They encourage a creative co-design approach to enable meaningful participation by all key stakeholders: an approach based on the principles of service design [26] and the UK Design Council’s *double diamond* model of the design process [27]. The approach involves divergent and subsequent convergent thinking phases to, in the first instance, open up the space of challenges before honing the problem definition and then to explore potential ideas before focusing on a workable solution to the problem in hand. If all these processes are done with the involvement of all key stakeholders, the approach becomes coproductive by definition [26]. The explicit use of creative methods in coproduction addresses many of the challenges of coproduction, namely imbalances or deficits of power, language, trust, and time. It fosters an inclusive nonhierarchical environment [28] that allows all participants to be recognized as experts and produces results in the form of visible and tangible outputs owned by those who created them [29].

### Participants

We aimed to recruit up to 10 individuals in total to 2 co-design workshops, drawn from the sets of patients with lived experience of COPD and of health professionals working with this patient group. In addition, we aimed to recruit a further 10 patients to take part in subsequent video-based scenario testing. In more detail, eligible participants were to be either of the following:

- An individual with a self-reported diagnosis of COPD or chronic lung disease, medically stable, and with a self-reported experience of emotional symptoms in relation to their LTC. A diagnosed mental health condition was not an inclusion criterion for taking part; however, as noted previously, mental health conditions in patients with physical comorbidities are common and often undiagnosed.
- Eligible health professionals were those working with this patient group in some capacity such as a general practitioner (GP; the doctor who will be responsible for treating all common medical conditions and for referring patients for urgent or specialist treatment), mental health professional, specialist nurse, occupational therapist, physiotherapist, or pulmonary rehabilitation specialist.

Individuals with lived experience were identified through the local British Lung Foundation *Breathe Easy* support group. A researcher (KE) attended the support group sessions and presented the research to members, along with information sheets. Interested individuals emailed the study lead (KE) to sign up for the workshops. Our professional contacts from NHS Trusts in and around the Yorkshire region of the United Kingdom were used to obtain the health professional sample: a snowball sampling technique was adopted, with senior managers forwarding an invitation email to relevant staff.

### Data Collection

#### Workshop 1

The first workshop was run to consider initial user requirements for the prototype system. Activities were conducted in small groups with time for feedback and reflection after each activity. This workshop focused on gaining a shared understanding of the lived experience of COPD by:

- Mapping out *a day in the life* journey together with a participating health professional. Sticker icons were used as prompts to help tell each person’s story and *missing sentence* prompt cards (“I wish I had known that…”; “It would have been good to have…”; “If only…”; and “It helped to have…”) were used to help participants reflect on the positive and negative impacts of their physical and mental health on their day. This process addressed Objective (1), namely the elicitation and prioritization of problems in need of solutions.
- Showing participants examples of existing virtual artificial agents to critique. Mood boards were populated to define the ideal set of features for Ava and Avachat. The look, sound, format, and potential features of Ava and Avachat more generally were explored to address Objective (2), eliciting the operational requirements of the system.

#### Workshop 2

**Figure 1:**
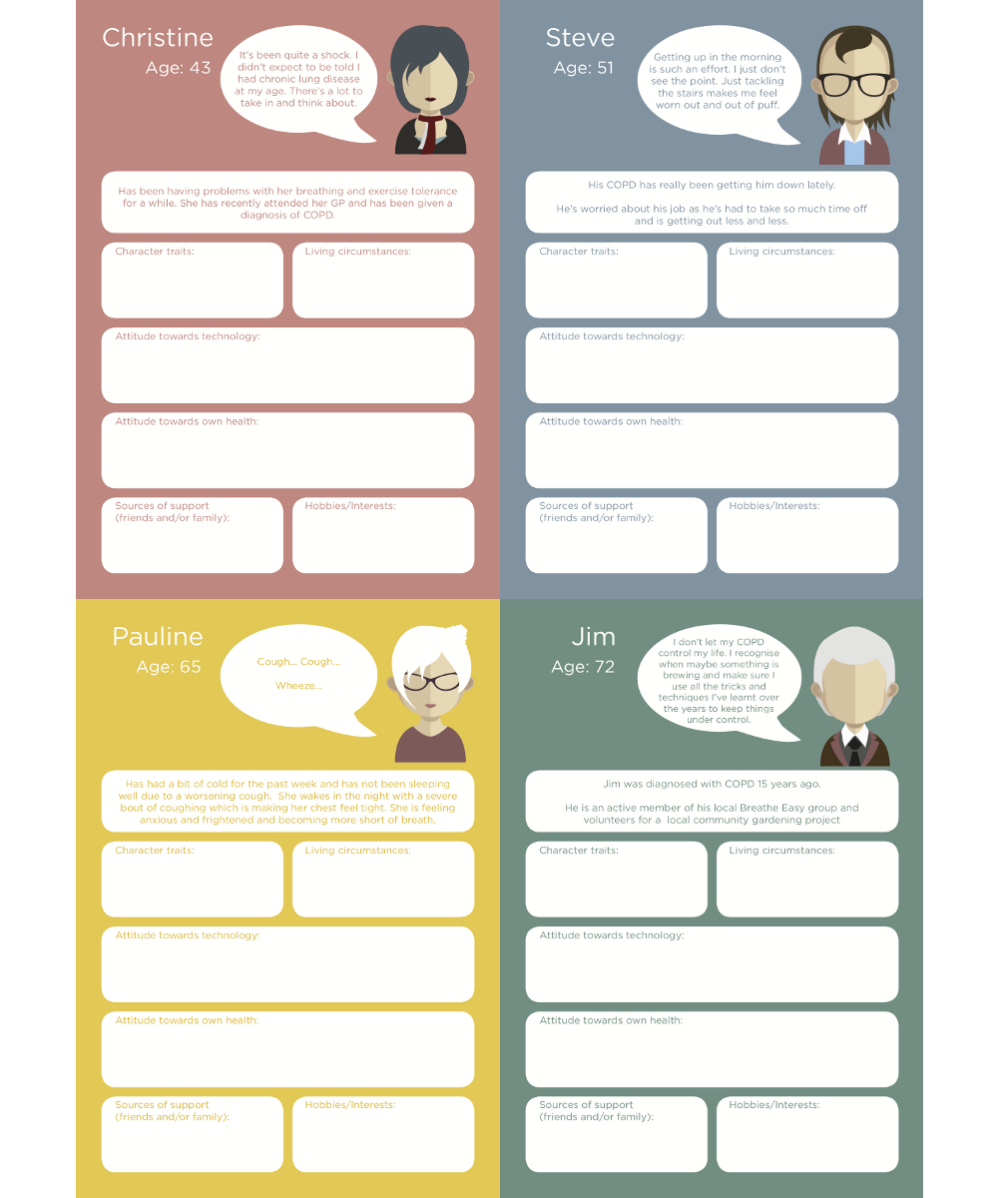
Persona worksheets. COPD: chronic obstructive pulmonary disease; GP: general practitioner.

**Figure 2:**
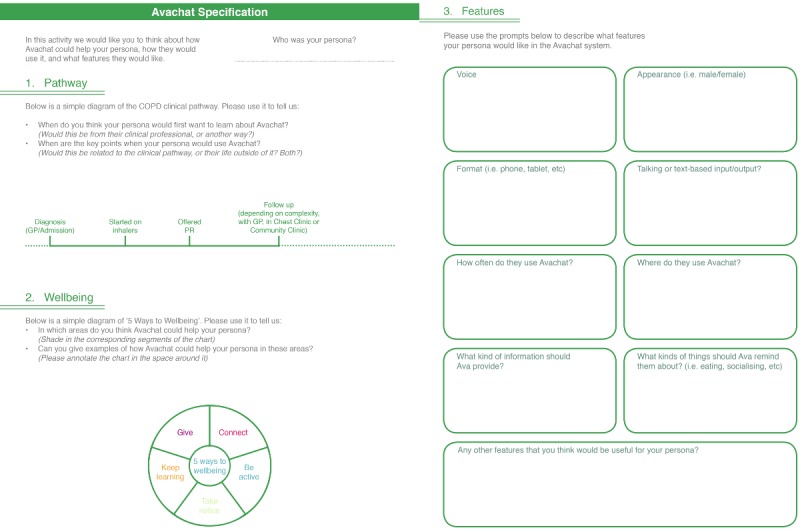
Vignette feedback sheets. COPD: chronic obstructive pulmonary disease; GP: general practitioner; PR: pulmonary rehabilitation.

The aim of this workshop was to design and develop how Avachat, in the guise of Ava, would deliver the core functionality identified in the first workshop. This would address Objective (2) eliciting operational requirements. Activities including the following:

- Adopt and elaborate one of a number of vignettes (Figure 1)—each corresponding to an archetypal person living with COPD—using techniques such as role play to allow participants to step out of their particular contexts and move toward a shared, empathic understanding of different potential users’ needs, experiences, behaviors, and goals.
- The vignettes then informed the development of *scripts* or sample interactions with Ava in a particular scenario, each related to one of the core functionalities of the system. Each script constituted an essentially linear dialogue, with some limited branching to explore particularly problematic or interesting alternatives. The scripts helped refine the nature of the support that would be expected of the system as well as define aspects such as safeguards (in what circumstances it was advisable and acceptable to deviate from the script to alert a GP or caregiver, for instance), the style of language and speech that should be adopted by Ava.
- Feedback on the vignettes (Figure 2) was used to gather further information for the Ava prototype development.

### Video-Based Scenario Testing of Acceptability

Following the workshops, we incorporated some of the more achievable user requirements and content into a semiautonomous prototype Avachat system. On the basis of the scripts developed during Workshop 2, we also developed a short screenplay that introduced Ava and the concept of Avachat and depicted how it might provide support to its user in typical domestic situations. This screenplay was filmed in a living laboratory facility, with a volunteer person with COPD taking the role of the user and simulated interactions with Ava (with an offscreen human operator acting as a *Wizard of Oz* to provide appropriate responses). This material was edited into a short video (under 8 min), which was then used as the basis for scenario testing with a sample of patients to determine acceptability of the application and content. We explored participants’ views and opinions and asked them to self-complete the System Usability Scale (SUS) questionnaires [30]. The measure has 10 items on usability, complexity, need for support or expert knowledge, integrity, and consistency. Each question is rated on a Likert scale from 1 to 5 (strongly disagree to strongly agree).

### Data Analysis

Workshop activities were conducted on paper and were additionally captured in photograph and video format. Data from Workshop 1 on the challenges of daily living were analyzed using content analysis techniques [31] to determine the scenarios around which to develop the core functionality of Avachat. In addition to providing further operational requirements, outputs from Workshop 2 were synthesized using thematic analysis [32] to develop a set of 4 vignettes (case scenarios) that would serve as exemplars for interactions with Ava. We used 2 of these in the screenplay that was filmed and then during the video-based scenario testing. The discussions from the scenario testing were transcribed verbatim and presented as collections of conversation around a priori themes of design, functionality, and content. The completed SUS questionnaires were scored as stipulated by the developers of the instrument. Higher scores are indicative of a more user-friendly system. Although SUS was originally developed to compare alternative systems and their interfaces for performing the same task, its developers argue that, as some sort of absolute rating, a score of 68 represents an average score across all systems.

## Results

### Workshop 1

#### Characteristics of the Sample

The workshop was run in July 2017 and lasted 5 hours. Overall, 5 individuals with a diagnosis of COPD and a carer of an individual with COPD agreed to attend (n=6), of whom 5 attended (male n=2 and female n=3), with 1 dropping out because of ill health (male). Participants had a median age of 82.5 years (range 69 to 86 years) and had been diagnosed with COPD for an average of 10.5 years. No medical data on the stage of COPD were available. One participant was currently using oxygen daily. All participants had previously attended pulmonary rehabilitation courses. A total of 6 health professionals attended, all female, working across general practice, secondary care respiratory services, and specialist mental health care.

#### Activities Data

##### A Day in the Life

**Figure 3:**
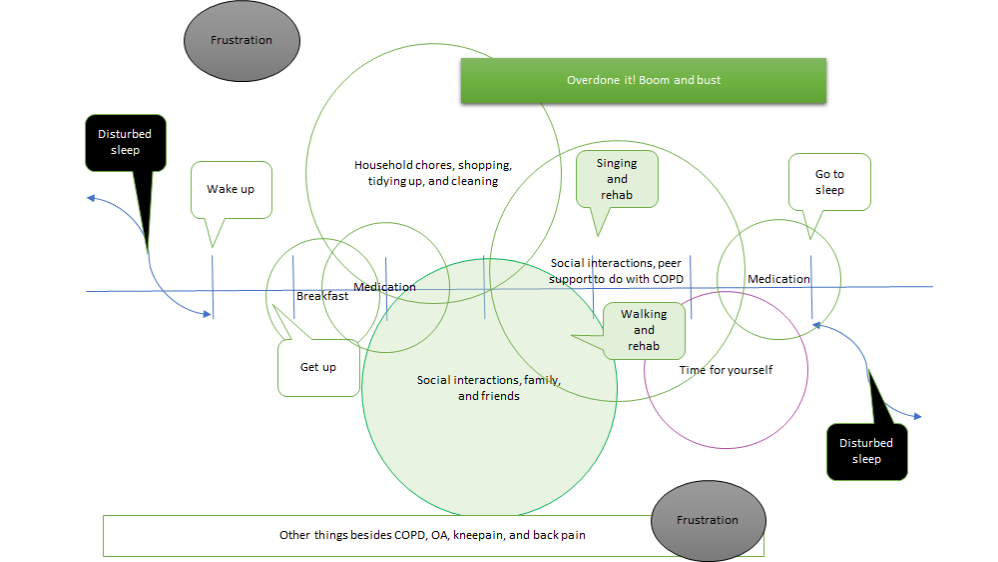
A day in the life of a chronic obstructive pulmonary disease patient. COPD: chronic obstructive pulmonary disease; OA: osteoarthritis.

All participants were medically stable. None of the participants disclosed COPD exacerbations or acute mental health problems. Conversation focused on ways in which they prevented low mood and social isolation. From the *a day in the life* exercise, the group identified positive and negative aspects of their lives. Figure 3 shows the range of activities described by participants. Household chores and social interactions occupied the majority of midday activities for participants. Evenings were quieter, with the group stating that they rarely went out of the home in the evening, often feeling very tired as the day progressed. However, the group reported that at times they lacked *motivation* (intrinsic or extrinsic) to follow through on planned household chores or social activities. Sleep was often interrupted at times because of their medical condition or associated medication (frequent need to urinate in the night, leg cramps, and breathing difficulties). Episodes of low mood were reported, particularly low mood on waking.

##### Missing Sentences Task

The missing sentences task highlighted that additional support and *information provision* in the early stages of the disease would have been beneficial:

It would have been useful to have been assigned to the respiratory nurse earlier on in my treatment, [to provide] general advice on medication/holidays/ support.

I think there should be more info for newly diagnosed patients of what services are available.

Members of the group felt that advice and support should be *informal* and that, ideally, they would have liked one-to-one support:

I think that information given by professionals should be less professional [in style] and more humanised.

If only there were individual staff for each patient to be around whenever needed.

In addition to this, the need for support in *crisis* situations or during acute exacerbations was highlighted:

I think people should be able to get more instant support, especially when feeling stressed.

I needed more support when my husband had a severe exacerbation/ panic attack. I called 999 [the UK emergency telephone number]. I need some strategies.

Carer

I wish I had someone around to help me when it [breathlessness] hits you.

Additional data from the missing sentence activity in Workshop 1 were related to the techniques that participants used to maintain or increase their *emotional well-being*. These resembled the New Economics Foundation’s *Five ways to wellbeing* [33] and were included in the Avachat scripts (Table 1).

**Table 1:** The *Five ways to wellbeing* and the data to support its application in Avachat. The terms in italics are the 5 recommended actions to foster one’s own well-being.

Components of well-being	Evidenced from the missing sentence activity
*Give....;* Your time, words, presence	“I have always been busy, driving the bus for the local care centre, being in the buddy system to support people....sitting in the clinics to encourage people to sign up for pulmonary rehab”; “....choir, breathe easy treasurer, meetings etc. But I am glad I do it. The benefits are wonderful”
*Be active....;* Do what you can, enjoy what you do, move your body	“I enjoyed the feel-good factor after exercising”; “I think GPs should introduce exercise as a treatment option earlier”; “I feel much more positive, healthy and confident since starting pulmonary rehab and breathe easy”
*Keep learning....;* Embrace new experiences, take opportunities	“There is a video now to help people with COPD understand more about the service—this needs sharing”; “I think there should be more info for newly diagnosed patients of what services are available”; “It was good to join the breathe easy choir and enjoy all the songs we sing from the shows etc. Good for the lungs and great for the spirits”
*Connect....;* Talk, listen, share, feel connected	“I enjoy hearing the positivity of the people with COPD—and how well they cope with what can be a difficult condition” (health professional); “It’s good to get perspective of other people’s experiences”; “I try and see someone every day, at the shops, playing bridge, at my friend’s for dinner....”
*Take notice....;* Notice small things	“I think people should carry on doing things. I like listening to the radio and watching the garden, the plants, the grass....I work hard to keep it nice. I’m in no rush”

#### Outputs From Workshop 1

##### Care Pathway Scenarios

The data generated from the *a day in the life* task and the missing sentences suggest 4 scenarios in which Avachat could support patients. These are at the time of diagnosis (*information provision*), during acute exacerbations (*crisis support*), during periods of low mood (*emotional support*), and for general self-management (*motivation*). Ideas for other features of the system included reminders for medication and appointments, and providing access to clinical results.

##### Avatar User—Specifications

**Figure 4:**
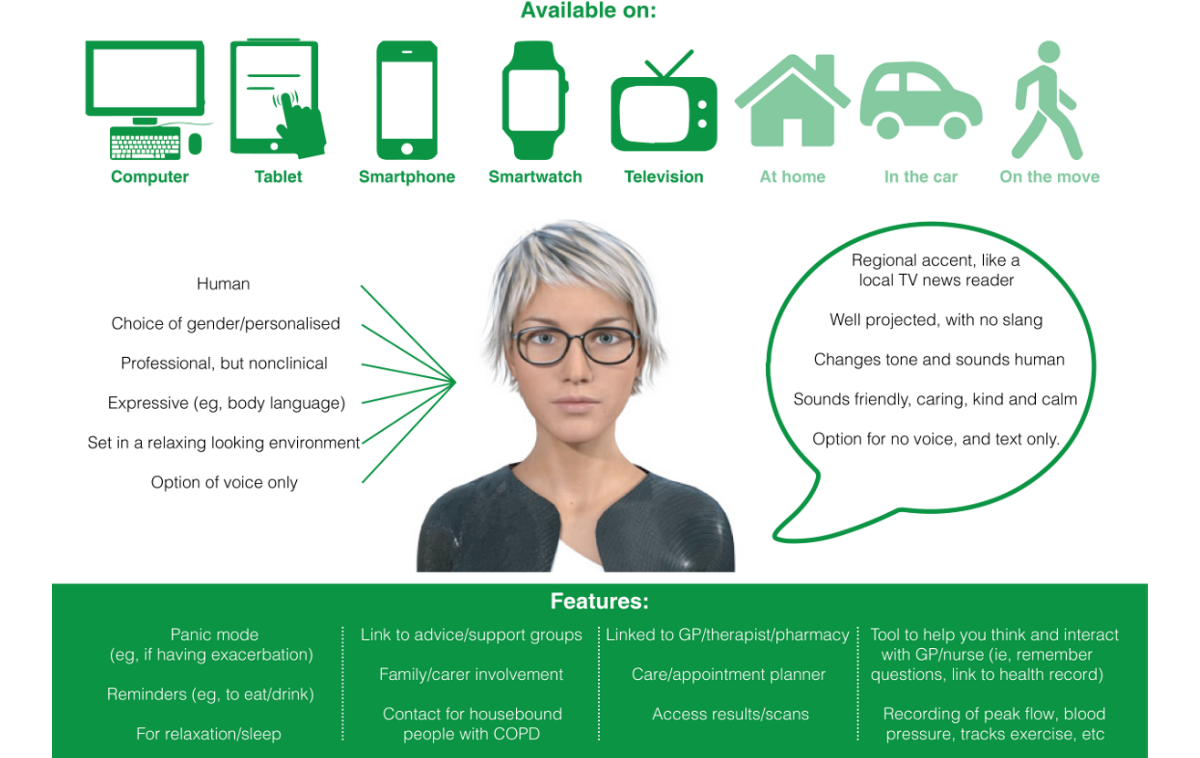
User specifications for Avachat (avatar graphic shown courtesy of Bot Libre). COPD: chronic obstructive pulmonary disease; GP: general practitioner.

Figure 4 illustrates the data generated from the critiques of existing artificial agents and mood board activities that participants undertook in Workshop 1. It shows the look, sound, format, and potential features of Ava that were considered important to the participants. Participants stated that they would like Ava to be multimodal and accessed via connected consumer devices such as their computer, tablet, mobile phone, or television. They wanted it to be recognizably human in appearance, to be able to personalize Ava to some degree (eg, to alter its *gender* and accent) and to have options for voice- or text-only communication. Participants spoke about the desire for a conversational discourse but also education provision (the predominantly one-way relay of information).

#### Script Content for Avachat

Draft scripts were developed for an introduction to Ava and the 4 scenarios. The content for the scripts was derived from clinical information (British Lung Foundation patient literature), behavior change theory (motivational interviewing), emotional well-being advice (New Economics Foundation’s *Five ways to wellbeing*), and peer-driven content (participants). This content was used as a basis for further elaboration of the scenarios by the participants in Workshop 2.

### Workshop 2

#### Characteristics of the Sample

The workshop was held in October 2017, and 5 patients who attended the first workshop also signed up for this second workshop. Of these, 4 attended (male n=2 and female n=2), with 1 drop out because of ill health (male). The median age of the participants was 73 years (range 66 to 80 years), and the average duration of diagnosis was 11.2 years. No data on comorbidities or stage of COPD were collected. Overall, 4 health professionals attended, all of whom had also attended Workshop 1, and all were female, working across secondary care respiratory services and specialist mental health care.

#### Activities Data

##### Scenarios

**Figure 5:**
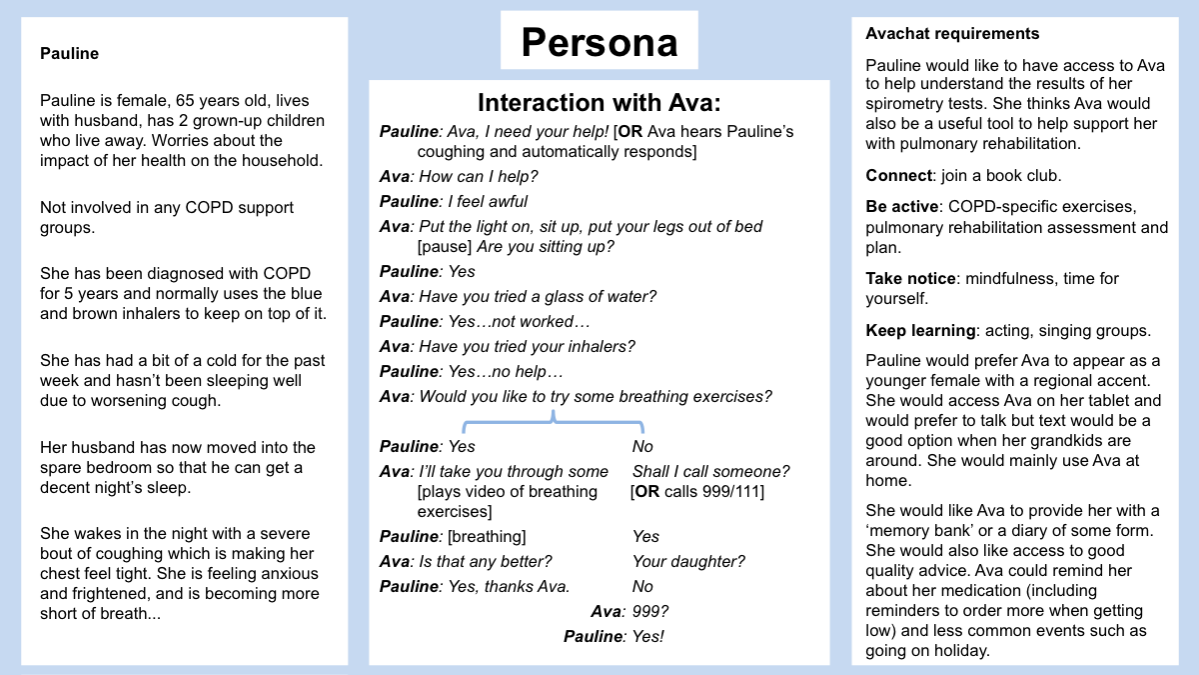
Persona crisis support. COPD: chronic obstructive pulmonary disease; 999 and 111 are the UK telephone numbers for life-threatening and non-life-threatening emergencies respectively.

Scenarios for exploration using vignettes included at time of diagnosis (*information provision*), during acute exacerbations (*crisis support*), during periods of low mood (*emotional support*), and for general self-management (*motivation*). Figure 5 illustrates the content of a persona focusing on the crisis support scenario.

#### Output from Workshop 2

##### Technical Development

We developed a responsive interface that presented Ava to the user via a variety of devices and allowed spoken or written interactions with the system. In addition, there was a second interface intended for a remote human *wizard*, hidden from the user: this interface would allow an interaction episode to be initiated and subsequently would display the user’s utterances and allow the wizard to choose or type an appropriate response and then transmit it to the user interface, where it would be both spoken aloud and displayed onscreen. (It was not considered necessary at this stage to handle more than 1 simultaneous user: we assumed there would be 1 user and 1 wizard per Avachat system.)

The scripts developed during the workshops were encoded internally as simple state-transition models that were used to present preformulated response alternatives as the user and Avachat proceeded through the script. If the user deviated from the main narrative of the script in a way that had not been envisaged, the wizard could fall back on a default *safeguarding* script, involving an emergency call to a relative or for an ambulance, but the wizard was otherwise left to his or her own devices. In this manner, the encoded scripts represented much of the Avachat system’s *knowledge* about the support that should be offered to users and, more implicitly, about appropriate ways of offering this support and interacting in an appropriate manner with the users.

Both user and wizard interfaces were implemented for Web browsers (using JavaScript). In addition to providing portability (the interfaces run on any computer with a modern browser installed), this meant that the underlying data communications between the 2 could be facilitated with the use of the internet and conventional HTTP protocols. Avatar graphics and animations for a range of different characters were licensed from a commercial company (Bot Libre) [34]. An initial implementation in Java provided a bespoke Web server for connecting the interfaces as well as the underlying script-processing mechanisms for the wizard. This effectively allows the wizard system and Web server to be run from a single laptop for maximum physical portability, allowing the system to be demonstrated in a variety of contexts. However, after the first workshop, and for greater convenience, the system was reimplemented in Python to run on a conventional, fixed-internet protocol (IP) Web server hosted at the University of Sheffield, which ensured that the system was always available and required a minimum of technical knowledge and time to run. In addition, in the course of reimplementation, and again for convenience, the speech recognition facilities of the system were changed from being based on the open-source Kaldi toolkit [35] to using those built into Google’s freely available Chrome browser.

Although this development process resulted in a system that was functional, in practice, unless the user conformed quite closely to one of the developed scripts, a significant amount of input was required of the wizard. Moreover, as it was to be expected that this input would often be health related, unless the wizard had experience of the LTC (as either a person living with the condition or as a health care professional), there was every likelihood that incorrect information or unhelpful (or even unsafe) advice might be shared. As a consequence, rather than attempting *live* interactive sessions with users of the system to explore acceptability, it was instead decided to use a video showing a carefully plotted *safe* interaction.

#### Wizard of Oz Video-Based Scenario Testing

As described above, we filmed 2 of the elaborated scenarios with a Wizard of Oz implementation of Avachat: during acute exacerbations (*crisis support*) and for general self-management (*motivation*). The film can be viewed in Multimedia Appendix 1. We then screened this video with 8 patients who had a diagnosis of COPD or were a carer of a spouse with COPD: 5 males and 3 females. Overall, 3 of these participants had been part of the co-design process, 5 were novel and new to the concept of Avachat. The median age of the group was 71 years (range 56 to 86 years). The stage of COPD was not reported; however, 1 participant was prescribed oxygen daily. No other comorbidities were reported. All but 1 of the participants used both a smartphone and tablet computer/laptop in their homes. After viewing the film, participants discussed the acceptability of Avachat with regard to design, functionality, and content.

##### Design

All members of the group apart from 1 reported generally positive feedback on the look and feel (and sound) of the Ava character:

I can’t believe it really. Impressive. (I can’t believe)...that she’s not real.

Participant 1

Nice looking girl isn’t she.

Participant 2

She says she’s here for you—like she will help, doesn’t talk down to you.

Participant 3

I think she’s lovely. I think she would say the right things and be helpful, just based on the way she looks.

Participant 1

The inferred informal interaction with Ava, as demonstrated in the film, was mentioned, as was her role as a companion as opposed to health professional:

I like that she says she’s not a health professional, more of a companion or assistant but she has information to help.

Participant 3

There was some clarity needed on whether Ava was a real doctor or an advisor:

She’s not medically qualified is she—she just gives advice?

Participant 4

But she says that at the start doesn’t she, that she isn’t a human or doctor, that she is giving medical advice but not like a real doctor.

Participant 1

One participant reported that the technology was not sophisticated enough to appear *real* to them; however, another member of the group felt this could happen with more traditional technologies:

I find it off-putting that her mouth doesn’t match her words.

Participant 5

Yes but you can get that on the TV even.

Participant 2

##### Functionality

Participants’ responses supported the workshop findings that end users would like Ava to be multimodal:

I like her on a tablet.

Participant 3

What if you haven’t got one? How about a TV?—a lot of people have a TV, and in the bedroom.

Participant 4

I like the laptop because someone else might be using the TV.

Participant 1

Can’t you use any? Any at any time—phone, TV, tablet, laptop?

Participant 2

If you were housebound and not up to date with technology then TV would be the most useful and easiest to use and in the bedroom.

Participant 5

Out and about, it’s good to have somethings that’s mobile.

Participant 2

It could be something that you wear—like on your wrist like a Fitbit—instant access. Then she is there whenever you need her.

Participant 5

Some aren’t interested even bit one bit in technology. It would have to be TV for them

Participant 8

Choice is good—individual.

Participant 6

In addition, 3 female participants discussed being about to talk to Ava and use both speech and text to communicate:

How about like the Amazon Alexa—bit like a radio: talk and it talks back. They can do things for you—scary.

Participant 1

Once you’ve seen her on an image it would be okay to just have the voice—I would like both.

Participant 8

If you could take her places—like to the doctors’ she could record what is said and help you and then you could talk to ava about what was discussed. We’ve seen her talking but you text her—that would suit me more than anything.

Participant 3

I can’t text so we need options.

Participant 7

Internet-of-things applications were touched upon by 3 participants:

Oh look, she said the air quality in the room is poor and humid, that’s great to know.

Participant 5

We could have all sorts attached, oximeter, to check oxygen levels.

Participant 1

If Ava could be wired into someone’s home to check you are okay, to listen to the coughing...could you have a pop up on your phone to ask if you are okay “do you want to speak to me?”

Participant 5

I like that she can see the temperature, humidity in the room etc...It helps you figure out why you might be coughing.

Participant 6

Moreover, 2 members of the group liked the idea that Ava could encourage you to call emergency services when appropriate:

People are scared of ringing the emergency services.

Participant 5

Ava could say you need to ring emergency services but sometimes people just need to be told to do that.

Participant 1

Participants were asked if they had any concerns relating to data, with respect to sharing data or where that data may go to once Avachat had received it. No members of the group voiced concerns in this area and 3 participants felt that sharing of data was commonplace in today’s society:

We’re not bothered, they [the government] know more about us than we do.

Participant 2

We are thinking it’s all safe.

Participant 6

It’s all on a database.

Participant 3

##### Content

When discussing the scenario of motivational support, the group thought this would be particularly useful for those living alone:

Brilliant that, a good idea.

Participant 4

Ava encouraged him to go out. If there is no one there to push you, you feel ill and your mood drops you don’t want to know. It can happen quite quickly.

Participant 5

As you get older you don’t have the social life. You don’t go out in the evenings even. You don’t go to the pub as you can’t drive and drink.

Participant 2

You always had someone around you in the past and small children can lift your spirits, but if you don't have that...mine [children] are miles away. If you need someone quickly...the nearest one is 3 hours away, but if you needed them, and they can’t always drop everything.

Participant 3

If you have a partner, they can encourage you.

Participant 6

It’s a lonely life if you don’t have anyone. That can be very depressing. It’s different if you have a partner, you can talk to them and they can talk to you and help in many ways.

Participant 2

One member also felt Ava could be useful for people who do not want to burden their partners or who do not feel comfortable doing so:

Your partner doesn’t always understand and you don’t want to burden them with things.

Participant 8

All participants reflected on the experience of acute COPD exacerbation and were particularly positive about this aspect of support from Avachat:

I were in on my own once, and I had this bad panic do, lucky my sister came, but I couldn’t get to the phone. I was right as rain at first. I had a coughing do and I must have panicked. It calms me down....the thought I could have someone to talk to, that’s good.

Participant 2

It certainly helps to know that someone is there.

Participant 8

After the screening, each participant was asked to imagine using the Avachat system in their own contexts, and, based on this, to complete a SUS questionnaire. Overall, 62% (5/8) of the SUS scores were above the score of 68 (with scores of 72.5, 75, 75, 80, and 85), with the other 38% (3/8) participants rating the system as follows: 52.2, 62.5, and 65. This gives the system an overall median rating of 73.75. 50% (4/8) of the participants strongly agreed with the statement “I think that I would like to use this system frequently,” and the other 50% (4/8) were unsure. Moreover, 88% (7/8) of the participants strongly agreed that the system was easy to use, possibly as the video shows a seamless use of the agent in practice. All but 1 of the participants strongly agreed that they would feel confident using the system. Views on whether they would need the support of a technical person to be able to use the system were split: half of the participants strongly disagreed that they would and half strongly agreed that they would. Again, with the statement “I needed to learn a lot of things before I could get going with this system,” 38% (3/8) participants strongly disagreed, 38% (3/8) strongly agreed, and 25% (2/8) were neutral.

## Discussion

### Principal Findings

Our aim was to explore the acceptability of both the form and the content of a computerized virtual agent with natural language capabilities intended to support self-management for people with comorbid LTCs and mental health problems. Through co-design with people living with COPD, we identified and prioritized 4 scenarios in which the system may be applied: at the time of diagnosis (*information provision*), during acute exacerbations (*crisis support*), during periods of low mood (*emotional support*), and for general self-management (*motivation*). The participants did not separate mental and physical health needs during the co-design process, although the generated content utilized psychological approaches to supporting patients to self-manage. Triangulation of clinically accurate information, behavior change techniques, emotional well-being advice, and peer-driven support appears to be the preferred content by patients. The results suggest that, for this small sample of older adults with comorbidities, it would be acceptable to receive both self-management support and support for acute exacerbations from an AI-based virtual agent.

The user requirements for the system were identified and many have been incorporated into a prototype system called Avachat. Specifically, we determined that the virtual agent must have the ability to understand and respond with both text and speech and must be accessible through a variety of domestic computing devices. The ability to interact with other digital services and domestic devices such as calendars, smart home technology, and medical peripherals is desirable. The system should be personalizable and its personification (Ava) should be emotionally expressive, and the system would be enriched by the ability to detect emotion (distress, fatigue, and irritation) in speech if it is to engage with users in a natural manner.

A blended approach to care has been shown to be most effective and acceptable for patients when managing both physical and emotional health [19]; however, as demand for support outstrips supply, it is crucial to explore what elements of person-to-person care could be provided using digital and AI-based systems. Indeed, a James Lind Alliance Priority Setting Partnership on digital technology and mental health, tasked with identifying the top 10 research questions posed by patients and clinicians, asks “Can common elements of therapy that come from person-to-person interactions be maintained with digital technology?” (priority 8) [36]. Our work speaks to this research question, in providing some evidence that a virtual agent could replicate certain aspects of traditional peer support incorporating behavior change techniques. With this patient group, such support is generally used to help patients better self-manage their conditions and decrease adverse health outcomes such as exacerbations, unnecessary emergency department attendance, and hospital admissions. Of course, patient outcomes are outside the scope of this work (and the capabilities of the current technology fall short of being able to provide such support in reality), so validation of the benefits for patients is work still to be done.

This empathetic ability of an automated system to detect and react appropriately to the nonverbal cues present in a person’s spoken utterances is likely to be crucial for the therapeutic relationship between agent and patient. Although not convenient for all people and in all situations, spoken language is the most immediate communication medium we possess and provides the basis for many conventional psychological or *talking* therapies. *Closed-loop* language interfaces with computers, which are able to both process the user’s speech and generate an appropriate spoken response, are becoming more sophisticated and are already found in popular consumer digital devices such as Apple’s Siri and Amazon’s Alexa (and, hence, are more and more a feature that users expect, at least in the English-speaking world; at the time of writing, the provision for other languages depends both on the number of speakers it has and the size of the consumer market they represent). Embodied virtual agents based on AI technology are already used in health-related software tools and apps, including a handful of mental health-focused products [37-42]. These have rarely been tested with samples of older adults (where most comorbidities are evident) [37,38]. A recent scoping review of conversational agents in clinical psychology [38] indicates that the ability of unconstrained natural language input capabilities for health-related purposes is an emerging field of research, although a computer agent that is able to maintain its side of a rich, open-ended dialogue necessary for the delivery of most psychotherapies is still some way off [43]. Even if not yet a practical proposition, we assume that users will soon expect the primary interaction modality of any autonomous therapeutic agent to be spoken natural language.

Participants in this study readily accepted Ava as a *human*, referring to it as *she*; they expressed a liking for her in terms of appearance, while the way in which she interacted with them suggests she *understands* the situation. Taken together, this suggests that the potential exists for the development of a relationship of sorts between human and autonomous agents, a suggestion supported by a recent study investigating the use of text-based AI to deliver psychological therapy [42].

### Limitations

Although based on input from those with lived experience of COPD and self-management, our findings were limited by the small sample of white, British, medically stable, regional participants recruited. None of the participants reported a current mental health problem but all referred to experiencing episodes of low mood or worry in the past. Furthermore, their existing engagement with the support groups through which they were recruited and their ability to attend the workshops suggest that they are representative of the type of patients who already self-manage their health. Although this meant that they could help us to identify the *best practice* with regard to self-management, it does raise doubts about whether the principles identified hold equally for those who cope less well or who are less experienced in self-management. In the next phase of our research, we will use more in-depth methods to engage hard-to-reach individuals, including those who are medically unstable, house bound, or from black and ethnic minority groups, those experiencing mental health conditions, or those for whom English is not their first language.

There was relatively little input from the health practitioners compared with patients during the workshops. This allowed the patient’s voice to dominate; however, it is not clear whether the health professionals agreed with or supported the final scenarios as a clinical priority or the content as best practice. Additional research is required to explore this stakeholder perspective and to place the system appropriately in the clinical pathway/patient journey or redesign that pathway, as appropriate.

This was not a software development project, although the research team did include software engineers, and as a result the amount of effort that could be assigned to development was limited. Our focus was the rapid development of an Avachat system that incorporated the user requirements as they emerged from the workshops; this enabled us to present the system to the participants to further refine their requirements and help elicit their wider opinions about the role of such technologies in their lives. This approach is promoted in the literature [38]. The system did not need to be fully functional (and, in any case, given the current level of technology available to us this would have been impossible); however, we wanted to produce a system that was as functional as possible and, hence, as accurate a reflection as possible of the short-to-medium-term potential of the technology. A purely imaginary requirements-gathering exercise for such a system would be difficult to constrain and would be likely to lead to a wholly impractical and unachievable specification. These considerations influenced many of the design and implementation choices for the system.

A point worthy of reflection is that rarely were the possible negative consequences of the technology discussed during the co-design and evaluation process. We did not raise these issues, preferring to allow the discussions to be participant-led and to specifically raise issues of duty of care, for example. Equally, consideration was given neither to the possibility of incorrect or inappropriate advice being given by the system nor to the wider ethical implications for the nature and standard of care that users would receive. It seems as though it was assumed that the system would be operate *perfectly*, without error or ambiguity and always in the best interests of the patient, and with no associated reduction in existing care. It is possible that, as consumers, we simply cannot fully understand the complex and dispersed data architectures and the complex distribution of roles, responsibilities, rights, and obligations that underlie newest digital technologies.

### Conclusions

Our research focused on co-designing the content and application of a conversational virtual agent to support self-management of physical and mental health comorbidities for patients with a diagnosis of COPD. We identified the underlying design concepts that would make the system useful and acceptable to the target population. Technological developments such as the automated delivery of therapy and health care support pose significant and difficult questions for our societies. Every new application of technology is accompanied by risks, and in this case, those risks touch upon the things we prize most dearly: health, social interaction, nature of work, and fair and appropriate distribution of limited resources. Although these developments can sometimes seem inevitable, the form that they take is not predetermined. Early stage co-design efforts such as those reported here represent a mechanism by which patient groups and their caregivers can have a voice in the process, and at a stage when not all the significant decisions have already been made.

Future research is planned to further develop and evaluate the system for a range of patient samples and care settings, in particular, crisis support. Clinical applications are broad. The benefits of a straightforward, low-cost system could include increased self-efficacy and self-confidence, reduced social isolation, and improved illness knowledge. Clinical gains could include fewer clinical exacerbations and a decrease in conventional health care service utilization, with an attendant reduction in costs.

## Appendix

Multimedia Appendix 1 Avachat film used for video-based scenario testing.

## Abbreviations

AI: artificial intelligence
COPD: chronic obstructive pulmonary disease
GP: general practitioner
LTC: long-term condition
NHS: National Health Service
NIHR: National Institute for Health Research
SUS: system usability scale
